# 15-PGDH inhibition in osteoarthritis: PGE_2_ skeletal interoception, cartilage regeneration and compartment-managed disease modification

**DOI:** 10.1038/s41413-026-00589-3

**Published:** 2026-10-15

**Authors:** Qiaojie Chen, Haijun Zhang, Yang Chen, Bo Zhang

**Affiliations:** Department of Orthopedics, Ningbo No. 2 Hospital, Ningbo, 315010 China

**Keywords:** Bone, Bone quality and biomechanics

## Abstract

Osteoarthritis is a whole-joint disease in which cartilage loss, synovial inflammation, subchondral bone remodelling and pain-related neural changes co-evolve, yet structural improvement does not reliably translate into pain relief. This Review centres prostaglandin E_2_ (PGE_2_) skeletal interoception in the translational framework for 15-hydroxyprostaglandin dehydrogenase (15-PGDH) inhibition. In skeletal interoception, PGE_2_ produced in mechanically stressed bone and osteochondral tissues is sensed by prostaglandin E receptor 4 (EP4)-positive sensory afferents, relayed to central circuits and translated into altered sympathetic norepinephrine output. Because 15-PGDH catabolises PGE_2_, its inhibition may prolong the persistence and spatial reach of this interoceptive cue while also acting locally on chondrocyte state. This combined local and interoceptive action offers a plausible mechanism for cartilage repair and pain modulation, but may also create compartment-specific risk when prolonged PGE_2_ persists in synovium or subchondral bone. We compare 15-PGDH inhibition with upstream synthesis blockade and receptor-specific strategies, and propose that development should be organised around three checkpoints: cartilage-enriched exposure, durability under mechanical load and structure–pain coherence. Current evidence supports cautious development rather than broad generalisation; clinical translation will require compartment-resolved pharmacokinetics/pharmacodynamics, EP-receptor attribution and early surveillance for synovial, osteochondral and pain-system liabilities.

## Introduction

Osteoarthritis (OA) is a heterogeneous, whole-joint disorder in which cartilage breakdown evolves alongside synovial inflammation, subchondral bone remodelling and activation of pain pathways.^1,2^ Therapeutic translation has been limited by persistent structure–symptom discordance: structural improvement, including cartilage repair, does not reliably produce durable pain relief.^3,4^ This disconnect reflects the multiple origins of OA pain, joint compartmentalisation and substantial placebo responses. Synovial fluid can redistribute soluble mediators between tissues, further weakening the link between structural change and symptoms.^5–7^

The population burden of OA is considerable. The Global Burden of Disease (GBD) 2021 analysis estimated that approximately 595 million people were living with OA in 2020 and projects prevalence to almost one billion by 2050, driven largely by population ageing and obesity.^2,8^ With longer lifespans and rising metabolic comorbidity, individual joints are more often exposed to combined risk factors, including cumulative mechanical load, obesity-related metabolic dysfunction and prior injury.^9^ This heterogeneity makes uniform treatment strategies increasingly difficult to justify. Large-scale genomics and endotyping may allow this heterogeneity to be used therapeutically through biologically informed patient stratification and pathway-matched interventions.^10,11^

Recent evidence has renewed interest in prostaglandin metabolism, particularly inhibition of 15-hydroxyprostaglandin dehydrogenase (15-PGDH), which prolongs signalling by slowing the catabolic clearance of prostaglandin E_2_ (PGE_2_) and other 15-hydroxylated prostanoids.^12^ The main preclinical impetus comes from a recent Science study in which 15-PGDH was induced in aged or injured cartilage, and its inhibition promoted articular cartilage regeneration while reducing OA-associated pain behaviours in mice.^13^ The proposed local mechanism is a shift in the transcriptional state of resident chondrocytes towards matrix-restoring programmes, consistent with single-cell and spatial transcriptomic evidence that cartilage contains multiple interconverting chondrocyte states.^14^ The central question for this Review is whether this local repair signal should also be interpreted through PGE_2_ skeletal interoception, in which joint-derived PGE_2_ is sensed by nerves and translated into altered sympathetic output.^15,16^

Skeletal interoception provides an integrative whole-joint framework. In this framework, PGE_2_ produced by mechanically loaded or injured skeletal tissues functions not only as a paracrine lipid mediator but also as an interoceptive signal that can activate prostaglandin E receptor 4 (EP4)-positive sensory afferents, engage central circuits and modulate sympathetic norepinephrine (NE) output to skeletal tissues.^15,17,18^ This pathway is directly relevant to OA because sympathetic tone, osteochondral remodelling and pain-related neural changes are part of the disease process rather than secondary observations.^4,19,20^

Within this pathway, 15-PGDH acts as a metabolic gate for the PGE_2_ interoceptive pulse. By degrading PGE_2_, 15-PGDH limits the amplitude, duration and spatial reach of PGE_2_ signalling; conversely, 15-PGDH inhibition can extend local PGE_2_ persistence at sites where PGE_2_ is already being produced.^15,21^ In OA, this may influence disease biology through two connected routes: a cartilage-intrinsic route, in which prolonged PGE_2_ biases resident chondrocytes towards matrix restoration, and an interoceptive route, in which PGE_2_ sensing by osteochondral sensory nerves reduces excessive sympathetic output and thereby supports cartilage homeostasis.^13,16,18^

We, therefore, organise this Review around a single causal chain: 15-PGDH inhibition prolongs endogenous PGE_2_; prolonged PGE_2_ engages receptor-defined local and interoceptive circuits; and these circuits may support cartilage regeneration when exposure is cartilage-biased and time-limited, but amplify synovial, osteochondral and pain-system liabilities when exposure drifts. We first define PGE_2_ skeletal interoception and the 15-PGDH gate, and then discuss cartilage state shifts, compartmental liabilities and development checkpoints.

## PGE_2_ skeletal interoception and the 15-PGDH gate

Skeletal interoception denotes neural sensing of the internal state of skeletal tissues and the feedback regulation that follows.^15^ In the PGE_2_-centred model, mechanical load, microdamage or osteochondral stress increases local PGE_2_ availability; this signal is detected by sensory afferents and relayed to central circuits, which then regulate sympathetic outflow to bone and joint tissues.^15,17,18^ For OA, this framework links three processes that are often considered separately: cartilage repair, osteochondral remodelling and pain-system activity.

This pathway can be framed as a bone/joint-to-brain-to-joint loop. Locally produced PGE_2_ activates EP4-positive sensory afferents; the signal ascends through peripheral and central neural circuits, and hypothalamic or related autonomic outputs adjust sympathetic norepinephrine release.^15,18^ In bone, this pathway has been linked to homeostatic bone formation. In articular cartilage, recent evidence indicates that increased local PGE_2_ skeletal interoception reduces sympathetic norepinephrine output and promotes anabolic renewal of the superficial zone.^16,18^

Within this loop, 15-PGDH functions as the catabolic gate. The enzyme oxidises PGE_2_ into less active 15-keto metabolites and thereby shortens local PGE_2_ pulses.^21,22^ Inhibiting 15-PGDH therefore does not create a new mediator; it prolongs the persistence and spatial reach of endogenous PGE_2_ produced during ageing, injury, loading or inflammation. In OA, this could strengthen a cartilage-supportive interoceptive signal by increasing PGE_2_ dwell time near EP4-positive sensory endings, while also maintaining direct EP2/EP4 signalling in chondrocytes.^13,16,21^

This duality helps explain why PGE_2_ signalling can be reparative in some OA contexts but liability-dominant in others. A time-limited, cartilage- or osteochondral-interface-biased PGE_2_ signal may favour matrix restoration and restrain excessive sympathetic drive; sustained PGE_2_ persistence in synovium or subchondral bone may instead reinforce inflammation, osteoclast-driven remodelling, angiogenesis, sensory nerve ingrowth and pain.^19,23,24^ The therapeutic question is therefore not how to increase PGE_2_ globally, but how to preserve a reparative interoceptive signal while preventing persistent liability-dominant signalling.

In this Review, compartment-managed disease modification refers to controlling where and for how long 15-PGDH inhibition extends PGE_2_ signalling. This principle structures the following sections on receptor attribution within the joint eicosanoid network, cartilage cell-state repair, interoceptive benefit-to-liability transitions and clinical trial gates.

## 15-PGDH inhibition within the joint eicosanoid network

15-PGDH inhibition is best viewed as a kinetic strategy, because it slows prostaglandin catabolism rather than introducing a new reparative mediator.^25,26^ 15-PGDH (encoded by HPGD) oxidises multiple prostanoids, including PGE_2_, into less active 15-keto metabolites, constraining both the duration and diffusion radius of signalling.^21,22^ In the interoception-centred framework, this enzyme also acts as a timing gate that determines how long a local PGE_2_ pulse remains available to chondrocytes, synovium, subchondral bone and EP4-positive sensory afferents. Structural work indicates that small-molecule inhibitors can stabilise 15-PGDH in a closed, catalytically inactive conformation, supporting the tractability of this catabolic node in prostanoid biology.^21^ Evidence for efficacy remains primarily murine: 15-PGDH expression increased in aged or injured articular cartilage, and systemic or intra-articular inhibition promoted cartilage regeneration and reduced pain behaviours across OA models.^13^

**Fig. 1:**
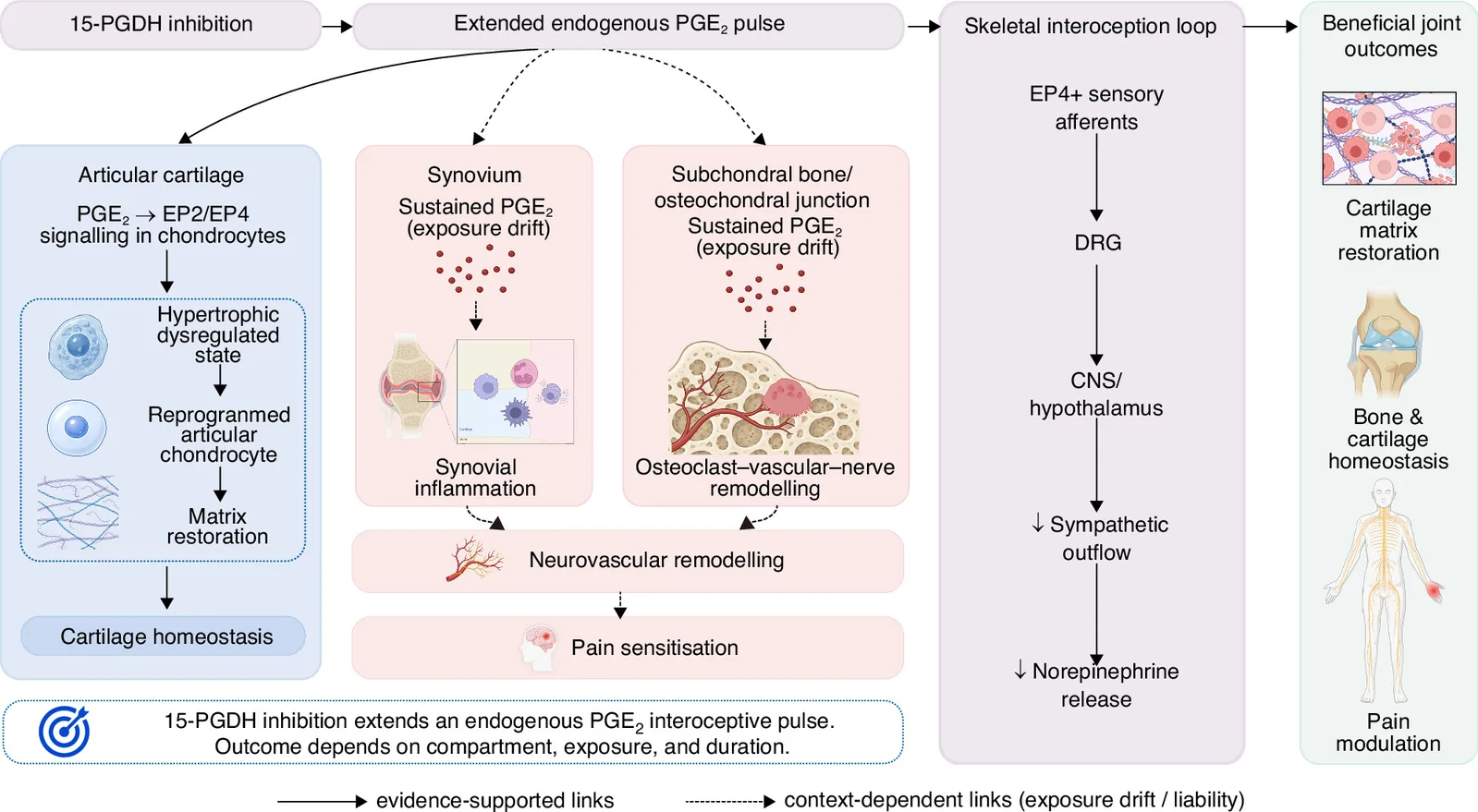
PGE_2_ skeletal interoception as the organising framework for 15-PGDH inhibition in osteoarthritis. 15-PGDH inhibition extends an endogenous PGE_2_ pulse rather than generating a cartilage-selective mediator de novo. When the extended signal is read in articular cartilage, PGE_2_–EP2/EP4 signalling may support chondrocyte state reprogramming and matrix restoration. The same PGE_2_ pulse can also be sensed by EP4-positive sensory afferents and relayed through dorsal root ganglion and central nervous system/hypothalamic circuits to reduce sympathetic outflow and norepinephrine release, contributing to cartilage matrix restoration, bone and cartilage homeostasis and pain modulation. If exposure drifts into synovium or the subchondral bone/osteochondral junction, persistent PGE_2_ signalling may instead promote synovial inflammation, osteoclast–vascular–nerve remodelling, neurovascular remodelling and pain sensitisation. Solid arrows indicate evidence-supported links; dashed arrows indicate context-dependent exposure-drift or liability links

In OA, this kinetic lever is relevant because the joint is pharmacologically compartmentalised and neurally coupled. Cartilage, synovium/vasculature, subchondral bone and sensory nerve endings differ in vascular access, diffusion barriers, receptor expression and interoceptive relevance (Fig. 1).^1,15,27^ PGE_2_ production is spatially patterned: inducible cyclooxygenase-linked pathways and terminal prostaglandin E synthases are preferentially engaged within inflamed and mechanically stressed microenvironments, so a single joint-averaged measurement can mask steep local gradients.^12,28–32^ For translation, the central question is which compartments sustain EP-receptor activation, at what amplitude and duration, and whether the signal is read locally by chondrocytes or systemically through skeletal interoception.^27,33,34^

A trial-relevant consequence is background cyclooxygenase (COX) inhibition. Many people with OA use NSAIDs or COX-2 inhibitors, which reduce upstream prostaglandin synthesis.^35–37^ Because 15-PGDH inhibition acts by extending the lifetime of endogenously produced PGE_2_,^21^ concomitant COX inhibition may reduce endogenous PGE_2_ availability and blunt both efficacy and mechanistic readouts; conversely, changes in NSAID use during trials can confound pain endpoints.^37^ Early studies should therefore pre-specify and stabilise NSAID regimens, or implement washout with defined rescue analgesia, and stratify randomisation by COX inhibitor exposure when washout is not feasible.^38,39^

15-PGDH inhibition differs pharmacologically from earlier approaches targeting the PGE_2_ axis. mPGES-1 inhibition reduces PGE_2_ production upstream, and a spontaneous canine OA study evaluated mPGES-1 and EP4-targeted approaches mainly as analgesic rather than cartilage-regenerative strategies.^40^ EP4-directed approaches have also produced mixed results: EP4 antagonism has reduced OA pain and promoted cartilage repair in some settings,^41,42^ whereas EP4 agonism suppressed chondrocyte hypertrophy and synovitis in surgically induced mouse OA.^43^ 15-PGDH inhibition is not equivalent to broader EP4 blockade. By prolonging endogenous ligand only where it is already produced, this approach may preserve reparative EP2 signalling and some context-dependent EP4 signalling that upstream synthesis blockade or constitutive receptor antagonism could remove. The same feature, however, can also amplify liability in compartments that are already rich in pathological PGE_2_.

PGE_2_ signals through four G-protein-coupled receptors (EP1–EP4; encoded by PTGER1–4).^35,44^ EP2 and EP4 couple to Gs to elevate cyclic adenosine monophosphate (cAMP), whereas EP1 and EP3 engage Gq/Gi pathways that can oppose or redirect these effects.^12,45^ This receptor diversity means that the direction of matrix regulation can change with cell type, differentiation state and extracellular milieu.^45,46^ In articular cartilage, chondrocytes predominantly express EP2 and EP4; the Singla et al. phenotype is consistent with the hypothesis that prolonged endogenous PGE_2_ biases chondrocytes towards a matrix-restorative programme, potentially through cell-state shifts rather than through anti-inflammatory effects alone.^13^

Although EP2 and EP4 both couple to Gs, they are not interchangeable.^45^ EP2-driven cAMP–PKA signalling has been linked to SRY-box transcription factor 9 induction and anabolic matrix outputs.^45,47,48^ EP4 signalling is more context dependent. In injured cartilage and subchondral bone, EP4 has been linked to metalloproteinase induction, hypertrophic-like differentiation, osteochondral remodelling and pain in certain settings.^12,23,41^ Yet EP4 agonism has also suppressed chondrocyte hypertrophy and synovitis in mouse OA,^43^ and broader chondrogenic contexts suggest that PGE_2_–EP4 signalling can contribute to homeostatic or anti-inflammatory responses.^49^ For 15-PGDH inhibition, EP4 should therefore be treated as context dependent rather than intrinsically deleterious. The relevant question is whether prolonged PGE_2_ drives cartilage-reparative or liability-dominant EP4 outputs in a given compartment, stage and exposure window.

Because receptor expression changes with tissue context and disease stage, prolonging ligand persistence is likely to engage a changing receptor environment rather than a fixed anabolic–catabolic balance.^21^ Peak PGE_2_ concentration is only one determinant of outcome. Time-integrated receptor engagement, local receptor abundance and the anatomical sites in which EP4 signalling operates will also shape whether the net effect is repair, inflammation or neurovascular remodelling.^19,50^ Receptor attribution is therefore essential for distinguishing cartilage-biased signalling from osteochondral and nociceptive liabilities.^13^

The same extended PGE_2_ signal can be maladaptive outside cartilage.^19,51^ In inflamed synovium, PGE_2_ signalling through EP receptors on synovial fibroblasts can sustain inflammatory mediator production.^19,52^ In subchondral bone, PGE_2_–EP4 signalling in osteoclasts couples remodelling to angiogenesis and sensory neuron ingrowth—processes closely tied to progression and pain.^23^ The osteochondral junction is increasingly recognised as a locus where neurovascular channels connect structural pathology to persistent pain even when cartilage thickness is an insensitive readout.^4,24^ The central liability is therefore not higher PGE_2_ per se, but sustained EP4-biased signalling in bone and perineural niches that can promote neurovascular remodelling and peripheral sensitisation.

These compartmental asymmetries are increasingly measurable. Single-cell atlases paired with spatial transcriptomics resolve cartilage, synovium and subchondral bone as interacting ecosystems and map prostanoid biosynthetic and catabolic programmes, together with PGE_2_-responsive circuits, to defined anatomical niches.^14,30,53^ Pain biology reinforces this view: peripheral prostaglandin tone is filtered through adaptable central networks^6^ and bone–nerve communication loops that can sustain symptoms independently of cartilage histology.^17,19^

Intra-articular exposure is rarely uniform: even direct injection can produce steep gradients in retention and penetration, so joint-wide signalling should be measured rather than assumed.^54–56^ Accordingly, we frame 15-PGDH inhibition as compartment-managed prostaglandin control and organise translation around three checkpoints: cartilage-enriched exposure with receptor attribution, mechanical durability and structural–pain coherence.

## Cartilage responses: local cell-state repair and interoceptive co-regulation

The translational appeal of 15-PGDH inhibition is not limited to increased cartilage thickness; it suggests that resident chondrocyte states can be biased towards matrix restoration and that this local repair signal may be reinforced by PGE_2_ skeletal interoception.^13,16^ In murine OA models, 15-PGDH inhibition promotes cartilage regeneration and reduces OA-associated pain behaviours.^13^ Recent interoception work provides a complementary mechanism: local PGE_2_ elevation can reduce sympathetic norepinephrine output and favour anabolic renewal of superficial-zone chondrocytes.^16^ The phenotype is therefore best framed as a combined local cell-state and neurohumoral response rather than a purely chondrocyte-autonomous effect.

Under this model, 15-PGDH inhibition may influence OA through interoception by extending the PGE_2_ signal available to EP4-positive sensory endings at the osteochondral interface. If this signal restrains excessive sympathetic drive, cartilage repair and pain-related behaviour could move in a favourable direction; if the same signal persists in synovium or subchondral bone, the response may shift towards inflammation, neurovascular remodelling or pain sensitisation.^15,16,20,23^ This benefit-to-liability transition explains why exposure geography and receptor attribution should be treated as central development variables rather than formulation details.

**Fig. 2:**
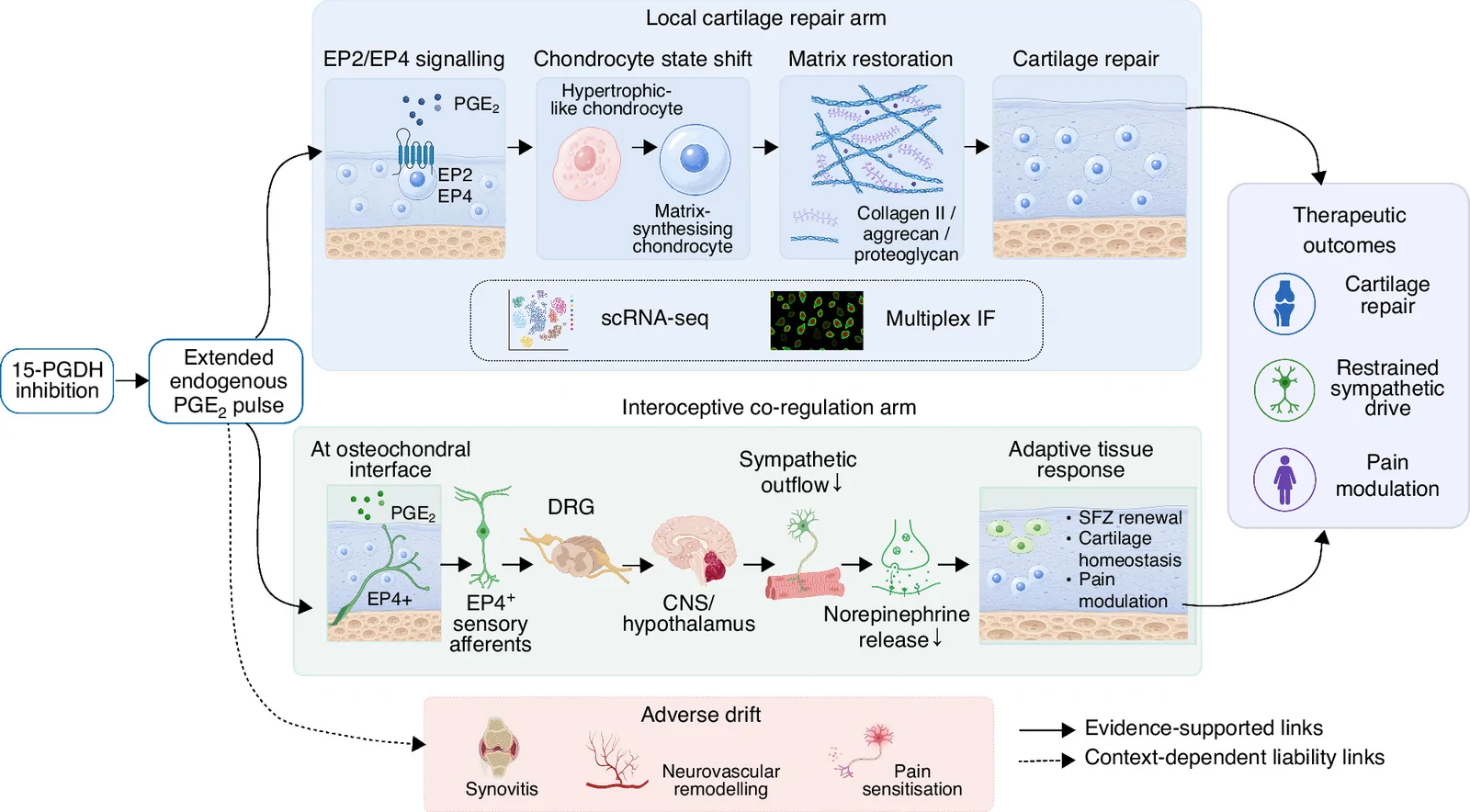
Proposed benefit chain linking 15-PGDH inhibition to local cartilage repair and interoceptive co-regulation. 15-PGDH inhibition is proposed to extend the endogenous PGE_2_ pulse and thereby support two connected therapeutic arms. The local cartilage repair arm links EP2/EP4 signalling to a chondrocyte state shift from hypertrophic-like chondrocytes towards matrix-synthesising articular chondrocytes, followed by collagen II, aggrecan and proteoglycan restoration and cartilage repair. The interoceptive co-regulation arm begins at the osteochondral interface, where PGE_2_ sensing by EP4-positive sensory afferents is relayed through the dorsal root ganglion and central nervous system/hypothalamic circuits to reduce sympathetic outflow and norepinephrine release, supporting superficial-zone renewal, cartilage homeostasis and pain modulation. Single-cell RNA sequencing and multiplex immunofluorescence support the murine chondrocyte state-shift step. Solid arrows indicate evidence-supported links; dashed arrows indicate context-dependent adverse drift towards synovitis, neurovascular remodelling and pain sensitisation. Receptor- and compartment-resolved validation will be required in human osteoarthritis contexts

The Science study provides a cellular anchor for this interpretation. Single-cell RNA sequencing and multiplexed immunofluorescence mapped the response to cellular identity, revealing a shift away from a 15-PGDH-expressing, hypertrophic-like chondrocyte population and towards an extracellular-matrix-synthesising articular programme (Fig. 2).^13^ These data support transcriptional reprogramming within resident chondrocytes, rather than expansion of a stem or progenitor pool, indicating that the intervention alters endogenous cell states rather than producing de novo cell replacement. This interpretation is consistent with cell atlases showing that diseased cartilage contains several discrete, interconverting chondrocyte states instead of following a single linear degenerative trajectory.^14,27^

Human data indicate that the cartilage states implicated in mice have patient counterparts, although evidence remains contextual rather than interventional.^57,58^ Multi-omics integration of human knee cartilage resolves inflammatory and prehypertrophic programmes as OA-critical states and proposes these as tractable targets for therapy and stratification.^30,57^ Single-cell atlases of infrapatellar fat pad (IPFP) and synovium further emphasise that peri-cartilaginous compartments are active signalling ecosystems with disease-linked programmes that can condition cartilage outcomes.^5,31,59^

Within this cellular framework, the murine state shift supports a testable prediction: if exposure and receptor engagement can be controlled, modulating eicosanoid clearance may bias conserved disease-associated cartilage states towards matrix restoration in situ.^13,14^ This mechanism extends beyond the broad claim that PGE_2_ can be anabolic; it specifies a kinetic mechanism acting on a resident equilibrium of cell states. This provides a route conceptually distinct from approaches reliant on transplantation, scaffolds or developmental morphogens.^3,12,27^ This cell-state interpretation does not imply that EP4 is uniformly reparative or uniformly deleterious. As discussed above, EP4 signalling is context dependent and may support either cartilage homeostasis/repair or liability-dominant programmes depending on compartment, disease stage and exposure window; receptor attribution therefore remains essential when translating these cell-state findings.^41,43,45,49^

At present, the efficacy and mechanistic case depends largely on a single well-controlled murine study.^13^ This signal now needs testing across OA contexts to define the conditions under which it is maintained or lost.

Two issues make such stress-testing essential. OA heterogeneity is mechanistic: age-associated disease, post-traumatic OA and chemically induced models differ in synovitis burden, subchondral remodelling and the timing and dominance of nociceptive inputs.^27,60–62^ In parallel, cartilage fate is shaped by the broader joint microenvironment: synovial inflammation can amplify, neutralise or even reverse a reparative cue, so synovitis should be treated as an active modifier of repair rather than as background variation.^1,63,64^ For 15-PGDH inhibition, systematic testing should therefore vary disease stage, inflammatory context, age, sex, exposure kinetics and delivery route, because signal persistence is part of the mechanism.^13,27,55,65^

DMOAD claims in OA increasingly require robustness across models, compartment attribution and paired structural–pain readouts. A voltage-gated sodium channel 1.^7^ study illustrates how multi-model genetic and pharmacological evidence can separate neuronal contributions to pain from chondrocyte-intrinsic control of progression while still showing organism-level benefit.^66^ Although the target differs, the evidentiary standard is relevant. Structural and pain outcomes are best treated as co-primary readouts measured under identical conditions rather than assembled from separate evidence streams.^67–69^

Previous disease-modifying osteoarthritis drug (DMOAD) development has shown that structural signals are not automatically clinically interpretable, and that pain can improve through systemic risk-factor modification without establishing disease modification.^1,27^ The STEP 9 semaglutide trial illustrates how sustained weight loss can yield substantial symptomatic and functional improvement without a direct claim to cartilage regeneration.^70^

Conversely, intra-articular programmes can generate signals that depend on baseline severity and endpoint choice; post-hoc analyses of lorecivivint datasets highlight how outcomes can be conditional on enrolment and outcome adjudication.^71,72^ Against this backdrop—and given large placebo and expectancy effects—early pain curves are an unreliable proxy for disease modification unless anchored to mechanism-proximal biology.^7,73,74^

The key question is whether a mechanistically grounded, joint-intrinsic intervention can shift structure and pain in the same direction by altering lesion biology rather than masking symptoms.^6,13^ The preclinical signal is notable, but not sufficient on its own. Translation will hinge on whether the chondrocyte-state shift persists under the stressors that typically derail OA programmes—compartmental heterogeneity, variable inflammation and pain mechanisms shaped by peripheral lesions and adaptable central circuits.^1,6^ Several methodological considerations and limitations should therefore be kept in view.

**Fig. 3:**
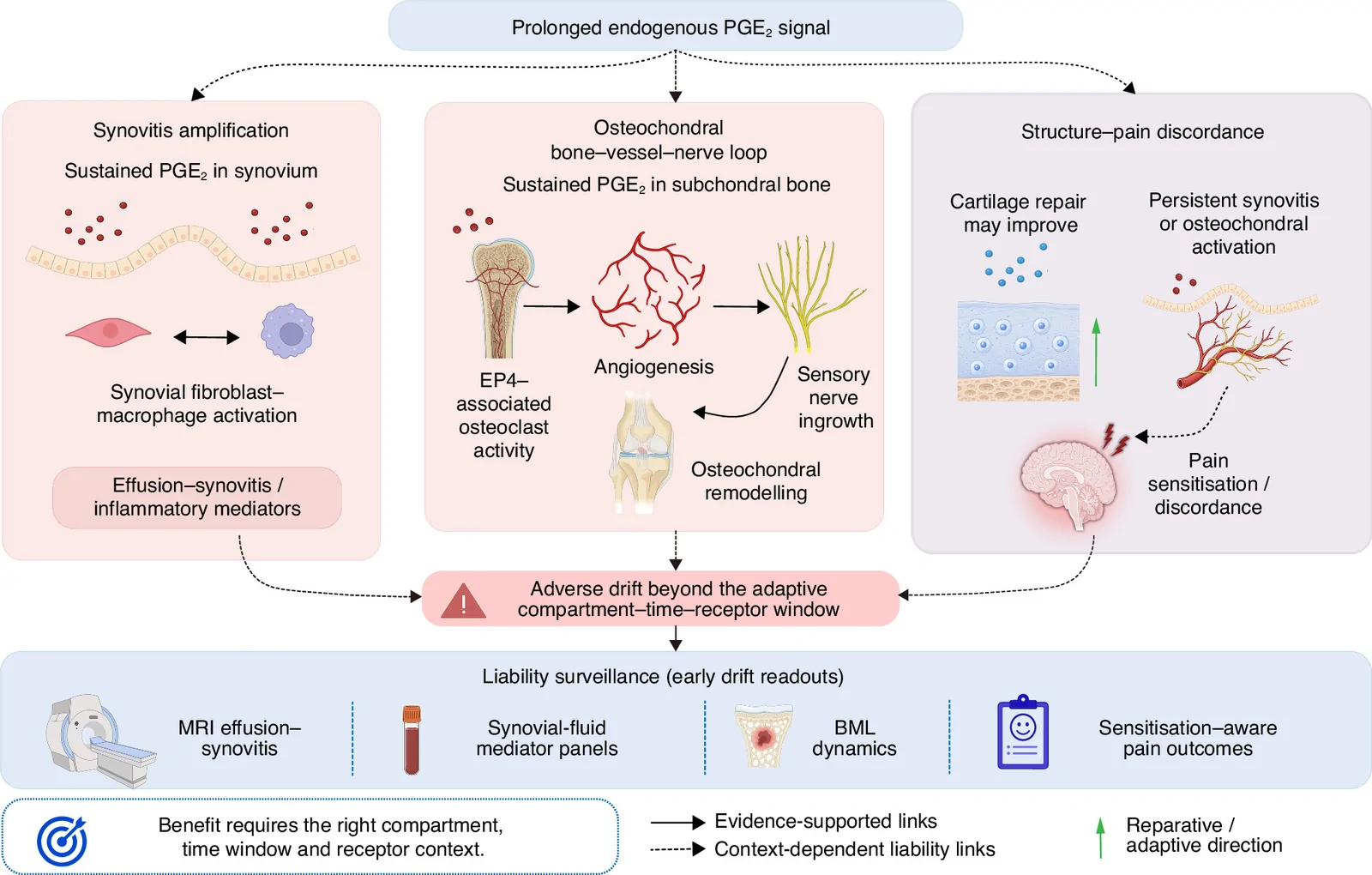
Benefit-to-liability transitions during prolonged endogenous PGE_2_ signalling. Prolonged endogenous PGE_2_ signalling can become liability-dominant when it persists outside the adaptive compartment, time window or receptor context. Sustained PGE_2_ in synovium may amplify synovial fibroblast–macrophage activation and effusion–synovitis/inflammatory mediators. Sustained PGE_2_ in subchondral bone may promote an EP4-associated osteochondral bone–vessel–nerve loop involving osteoclast activity, angiogenesis, sensory nerve ingrowth and osteochondral remodelling. A third failure mode is structure–pain discordance, in which cartilage repair may improve while persistent synovitis or osteochondral activation drives pain sensitisation. Early drift readouts include MRI-assessed effusion–synovitis, synovial-fluid mediator panels, bone marrow lesion dynamics and sensitisation-aware pain outcomes. Solid arrows indicate evidence-supported links; dashed arrows indicate context-dependent liability links; the green arrow denotes a reparative or adaptive direction

Most current evidence derives from murine injury-driven OA, and rodent cartilage is more regenerative than human cartilage.^3^ Intra-articular dosing does not ensure cartilage specificity; compartment-resolved pharmacokinetics/pharmacodynamics and EP-pathway engagement should therefore be quantified.^54^ Structural readouts should be paired with synovitis/BML surveillance and sensitisation-aware pain outcomes to adjudicate discordance.^7,75,76^ Durability and boundary conditions also remain unresolved, requiring longer follow-up, the stop–treat–observe (STO) durability framework proposed here, and mechanical stress-testing across endotypes and both sexes.^77–79^ These constraints motivate the compartment-specific liabilities discussed next (Fig. 3).

## Compartment-specific liabilities of PGE_2_ skeletal interoception and stabilisation

The preclinical rationale for 15-PGDH inhibition rests on its capacity to extend an endogenous PGE_2_ signal; the same property defines its testable liabilities.^80,81^ Prolonging PGE_2_ may support a reparative interoceptive loop when exposure is biased towards cartilage or the osteochondral sensory interface, but it may also sustain pathological signalling when exposure drifts into synovium, subchondral bone or nociceptive niches.^15,16,23^ In a compartmentalised whole-joint disease such as OA, risk should be framed as specific failure modes that emerge when the PGE_2_ interoceptive pulse becomes excessively persistent or is read in an inappropriate tissue–time window.^4,5,24^ Although we focus on joint compartments here, chronic systemic inhibition would add broader safety constraints, making exposure control and local delivery central to risk mitigation.

### Synovitis as an inflammatory amplifier

Synovitis varies across patients and disease stages, yet it consistently associates with pain and progression.^82,83^ The synovium is also an early-exposed compartment: lining and perivascular niches are readily accessible, and synovial fluid distributes soluble mediators across tissues.^5,63^ If 15-PGDH inhibition prolongs prostaglandin signalling here—via distributional bias or systemic exposure—synovitis may become an early pharmacodynamic marker of inflammatory engagement rather than cartilage-biased repair.^82,84^

Single-cell atlases strengthen this concern: synovium and IPFP form an immunostromal unit with disease-associated stromal and macrophage states capable of shaping cartilage biology and nociceptor sensitisation.^31,32,85^ Sustained prostaglandin signalling in this compartment could reinforce programmes linked to effusion–synovitis, vascular remodelling and inflammatory pain—features often enriched in symptomatic trial cohorts.^82,86,87^ Effusion–synovitis imaging and synovial-fluid mediator panels should therefore be treated as part of the causal chain that determines whether prolonged PGE_2_ signalling is interpreted biologically as reparative or inflammatory.^31,63^

### Osteochondral interoception: benefit-to-liability transition

When considered through PGE_2_ skeletal interoception, the osteochondral junction is not merely a liability compartment but a site at which benefit and risk diverge. Neurovascular growth, microdamage and remodelling at the cartilage–bone interface can sustain pain and progression with only weak correspondence to cartilage thickness.^4,24^ In the adaptive direction, local PGE_2_ is sensed by EP4-positive sensory afferents, relayed to central circuits and translated into reduced sympathetic output that supports bone and cartilage homeostasis.^15,16,18^ In the liability-dominant direction, persistent subchondral PGE_2_ can couple osteoclast activity, angiogenesis, nerve ingrowth and pain.

This bidirectional logic helps explain why PGE_2_ can appear both reparative and pathogenic. Chen et al. showed that osteoblast-derived PGE_2_ activates EP4 in sensory nerves to regulate bone formation by suppressing sympathetic tone.^18^ Wang et al. then extended this concept to articular cartilage, reporting that PGE_2_ skeletal interoception promotes anabolic renewal of superficial cartilage by tuning down sympathetic norepinephrine output.^16^ For 15-PGDH inhibition, this has a direct implication: by increasing the dwell time of endogenous PGE_2_, the intervention may strengthen a joint-to-brain-to-joint feedback signal in addition to altering chondrocyte-intrinsic EP signalling. Consistent with this neural relay model, recent primary evidence in ageing-related OA mice suggests that the adrenergic arm can itself be disease-promoting: β1-adrenergic receptor blockade attenuated OA progression, and sympathetic depletion ameliorated OA pathology.^20^

Yet skeletal interoception does not mean that more PGE_2_ is always beneficial. The Cell Metabolism review framed skeletal interoception as a general framework for bone homeostasis and pain, and recent OA-focused reviews have applied this idea to subchondral innervation, PGE_2_ biology and celecoxib.^15,19,35,43,88^ Mechanistic studies sharpen the boundary condition: elevated COX-2/PGE_2_ activity in subchondral bone can define a pathogenic OA subtype,^89^ and PGE_2_–EP4 signalling in subchondral bone osteoclasts can drive remodelling, angiogenesis and pain-associated nerve ingrowth.^23^ The translational implication is that 15-PGDH inhibition should preserve adaptive, cartilage-supportive interoceptive signalling while avoiding prolonged subchondral persistence that converts the same axis into a bone–vessel–nerve liability loop.

Bone marrow lesions (BMLs) make this liability clinically tractable. They represent biologically active subchondral domains linked to pain and structural deterioration,^75^ and single-cell profiling points to inflammatory, senescence-enriched immune landscapes with communication routes that could propagate cartilage damage.^53^ If 15-PGDH inhibition increases PGE_2_ persistence in these niches, BML dynamics, subchondral vascular markers and neurovascular readouts become part of the mechanistic safety assessment rather than optional adjuncts.

### Pain-system coupling and endpoint discordance

OA pain is multiscale: peripheral inputs from synovium, bone and the osteochondral interface are transformed by spinal and supraspinal circuits, allowing symptoms to persist even as structural trajectories change.^4,6^ Because articular cartilage is aneural, clinically meaningful pain is driven largely by non-cartilage compartments and their sensory couplings.^76^

PGE_2_ lies at the interface of inflammatory signalling and neuronal excitability, making it a plausible driver of structure–symptom discordance.^90^ Liability may therefore present less as overt adverse events and more as endpoint discordance: cartilage biomarkers improve while pain plateaus or worsens, particularly in sensitisation-enriched patients or those with osteochondral-dominant disease.^6,7,67^ Large placebo and expectancy effects further complicate early pain readouts.^7^

For development, a key liability is coupling failure: cartilage repair accompanied by persistent synovitis, osteochondral neurovascular growth or sensitisation-dominant pain. The murine evidence is notable because structure and pain improved in parallel.^13^ Whether this pairing translates to humans will depend on preventing sustained prostaglandin signalling from being interpreted as an inflammatory or neurovascular cue outside cartilage.

Together, these points identify controllable variables: exposure distribution and kinetics, receptor-pathway bias, osteochondral neurovascular surveillance and pain-endpoint design. These variables, and the failure modes they predict, motivate the checkpoints discussed next. The main liability modes and candidate readouts are summarised in Fig. 3. Key evidence and current gaps are summarised in Table 1.

**Table 1:** Evidence matrix for interoceptive benefit, liabilities and translational gaps

Evidence (selected refs.)	What it supports	What is missing
Anchor efficacy package: murine OA repair + analgesia^13^	Proof-of-concept that 15-PGDH inhibition can couple cartilage restoration with reduced pain behaviours; links phenotype to chondrocyte state redistribution by single-cell RNA sequencing and multiplex immunofluorescence.	Independent replication across OA contexts (age-, metabolic- and post-traumatic); compartment-resolved PK/PD and EP programme attribution; durability under load and after withdrawal.
Target tractability^21^	Supports feasibility of small-molecule 15-PGDH inhibition as a kinetic lever on prostaglandin clearance.	Joint-relevant selectivity and exposure window; demonstration that pharmacology delivers cartilage-biased engagement without synovial or osteochondral drift.
Human substrate for state-biasing and compartment context^31,32,53,57^	Human cartilage multi-omics and multi-tissue single-cell atlases define OA-critical chondrocyte programmes and peri-cartilaginous immunostromal ecosystems (synovium/IPFP and osteochondral/BML niches) with communication routes that can condition cartilage biology and pain.	Direct human evidence of compartment-specific HPGD and EP-receptor programme engagement and PGE_2_-response circuits; biomarker anchors that link target engagement to state shifts (repair) versus liability signatures (synovitis/BML activation).
Skeletal interoception and osteochondral liability evidence^15–20,23,53,75,88,89^	Supports a dual reading of PGE_2_ in OA. Bone- or osteochondral-derived PGE_2_ can engage an EP4-positive sensory nerve-central circuit-sympathetic axis that may favour anabolic renewal of superficial articular cartilage, yet sustained subchondral PGE_2_/EP4 signalling can also drive remodelling, angiogenesis, sensory nerve ingrowth and pain-linked osteochondral liability. This makes skeletal interoception the organising framework for benefit and risk, and makes BML dynamics and neurovascular markers mechanistically relevant surveillance readouts rather than optional exploratory measures.	Human, compartment-resolved evidence linking 15-PGDH inhibition to subchondral PGE_2_ persistence, EP4-positive sensory-nerve engagement and sympathetic output; thresholds for duration and amplitude at which interoceptive signalling shifts from adaptive/cartilage-supportive to liability-dominant; validated translational biomarkers integrating BML dynamics, neurovascular activation and pain-system coupling.
Comparator evidence across the PGE_2_ axis (mPGES-1 and EP4-directed strategies)^40–43,45^	Shows that interventions along the same axis can yield divergent outcomes depending on level and context: upstream mPGES-1 suppression and EP4-directed strategies have mainly been developed as analgesic or chondroprotective comparators, whereas both EP4 antagonism and EP4 agonism have shown benefit in different OA settings. This supports the argument that 15-PGDH inhibition is not equivalent to generic EP4 blockade, but rather a kinetic prolongation strategy that may preserve reparative EP2 and context-dependent EP4 outputs if exposure is controlled.	Direct head-to-head studies comparing 15-PGDH inhibition with mPGES-1 inhibition, EP4 antagonism and EP4 agonism under matched OA models, delivery routes and structure–pain endpoints; receptor-resolved attribution in cartilage versus synovium/subchondral bone; evidence defining when optional EP4-bias mitigation preserves repair versus removes necessary homeostatic signalling.
Exposure-control feasibility and delivery constraints^3,54,55,77,78,91^	Establishes that cartilage penetration, deep-tissue retention and residence time can be engineered (penetrant cationic carriers, cartilage-retentive nanoparticles and mechanically resilient depots); highlights constraints imposed by inflamed, enzyme-rich and mechanically loaded joints.	Integration of exposure-control platforms with 15-PGDH inhibitors; quantitative cartilage versus synovium/subchondral exposure and time-integrated EP engagement; confirmation that exposure control prevents synovial/osteochondral liability sampling over time and under load.
Benchmark standard for disease-modifying claims^66^	Illustrates expected evidence discipline: robustness across multiple OA models, compartment attribution, and paired structural and pain readouts under identical conditions.	For 15-PGDH: multi-model, sex-aware replication with co-primary structure–pain endpoints; predefined discordance handling and clear go/no-go gates.
Clinical interpretability guardrails^7,67,70,71,73^	Reinforces that pain can improve without disease modification; outcomes can be conditional on enrolment and endpoint choice; patient-reported outcome properties plus large placebo/expectancy effects can dominate early pain readouts and obscure mechanism.	Early clinical studies need mechanism-proximal biomarkers and liability surveillance (effusion–synovitis imaging, synovial-fluid mediator panels, BML dynamics), plus sensitisation-aware outcomes and pre-specified rules to adjudicate structure–pain coherence beyond placebo.

## Translational checkpoints for PGE_2_-stabilisation DMOADs

The preclinical evidence is notable because it links a joint-intrinsic intervention to both cartilage restoration and reduced pain behaviours.^13^ For translation, the key question is whether the PGE_2_ signal extended by 15-PGDH inhibition can be maintained within an appropriate spatial and temporal range in a compartmentalised, mechanically loaded and neurally coupled joint.^1,15,27^ Three checkpoints operationalise this challenge: spatial control, durability under mechanical load and structure–pain coherence.

### Spatial control: achieving cartilage-enriched exposure

Intra-articular delivery is not synonymous with cartilage specificity. Synovial lining and perivascular niches are readily accessible; synovial fluid redistributes mediators; and disease-dependent osteochondral microchannels can expose subchondral bone to intra-articular signals.^5,24^ The translational question is which compartments sustain target engagement, with what kinetics, and through which EP receptor programmes.

Compartmental distribution can, in principle, be engineered. Inflamed, enzyme-rich and mechanically active joints impose hard constraints on residence time, penetration and manufacturability.^3,77^ These constraints should be considered part of the therapeutic mechanism rather than a downstream formulation issue. Matrix-binding motifs, cartilage-retentive depots and penetrant carriers offer practical routes to tune residence and penetration while resisting rapid clearance.^55,78,91^ Cartilage-targeting cationic peptide carriers that achieve deep penetration and retention in large-joint post-traumatic OA illustrate that compartment bias can be designed rather than assumed.^54^

This checkpoint is therefore relevant to both efficacy and risk control. Because the osteochondral junction is pain- and progression-relevant, cartilage engagement should be demonstrated alongside an absence of early synovial or osteochondral drift.^24,92^ This requires compartment-resolved exposure and target-engagement measures and receptor-proximal signalling attribution, as PGE_2_ elevation without receptor-proximal context is biologically ambiguous in a pleiotropic system.

### Mechanical durability under load

Cartilage that appears repaired over weeks can fail over months to years because OA is shaped by mechanics as well as molecular cues. Cyclic loading, interface shear and fatigue can erase early gains while subchondral remodelling and gait adaptation evolve in parallel.^3,77,93^ A 15-PGDH strategy therefore requires evidence beyond histology or early imaging alone: repair must remain durable under physiologically relevant loading.

Durability should be tested through both experimental design and delivery strategy. Preclinical testing should include longer follow-up, STO windows, activity paradigms that stress repaired tissue, and models that better approximate human joint biomechanics. For delivery, this favours depots whose release kinetics remain stable under deformation; mechanically resilient soft hydrogels provide a concrete example in physically active joints.^91^

If the premise is endogenous cell-state biasing rather than cell replacement,^13^ persistence becomes the key readout: the state shift should survive return to normal use, integrate across the osteochondral unit and remain after withdrawal. This checkpoint is met only when gains are mechanically meaningful, sustained and not merely treatment-dependent transient phenotypes.^77,93^

### Coupling structural and pain outcomes

A DMOAD claim requires structural and pain outcomes to be interpreted together, because pain is not a passive readout of cartilage injury. Peripheral inputs from synovium and the osteochondral unit are filtered through sensitisation and brain circuits, allowing symptom and structural trajectories to diverge.^4,6^ Because cartilage is aneural, clinically relevant pain sits downstream of non-cartilage compartments and their sensory couplings.^76^ For PGE_2_ stabilisation—given its pleiotropy and proximity to nociceptor excitability—programmes should not advance on cartilage endpoints alone if pain biology shows discordant or adverse signals.

Structure–pain coherence depends partly on endpoint design. Preclinically, pain and structure should be measured as co-primary outcomes in the same animals under identical conditions, rather than inferred from separate experiments. In early clinical development, multimodal panels should adjudicate discordance—cartilage and subchondral imaging, effusion–synovitis readouts, and pain outcomes complemented by sensitisation-aware measures.^4,5^

Structure–pain coherence must also be interpreted against OA trial realities. Placebo and expectancy effects are substantial in OA and can obscure mechanism unless proactively managed.^7^ The goal is not perfect structure–pain alignment, but pre-specifying what discordance would imply—and which signatures (synovitis drift, osteochondral activation, sensitisation-dominant pain) should trigger redesign rather than escalation.

**Fig. 4:**
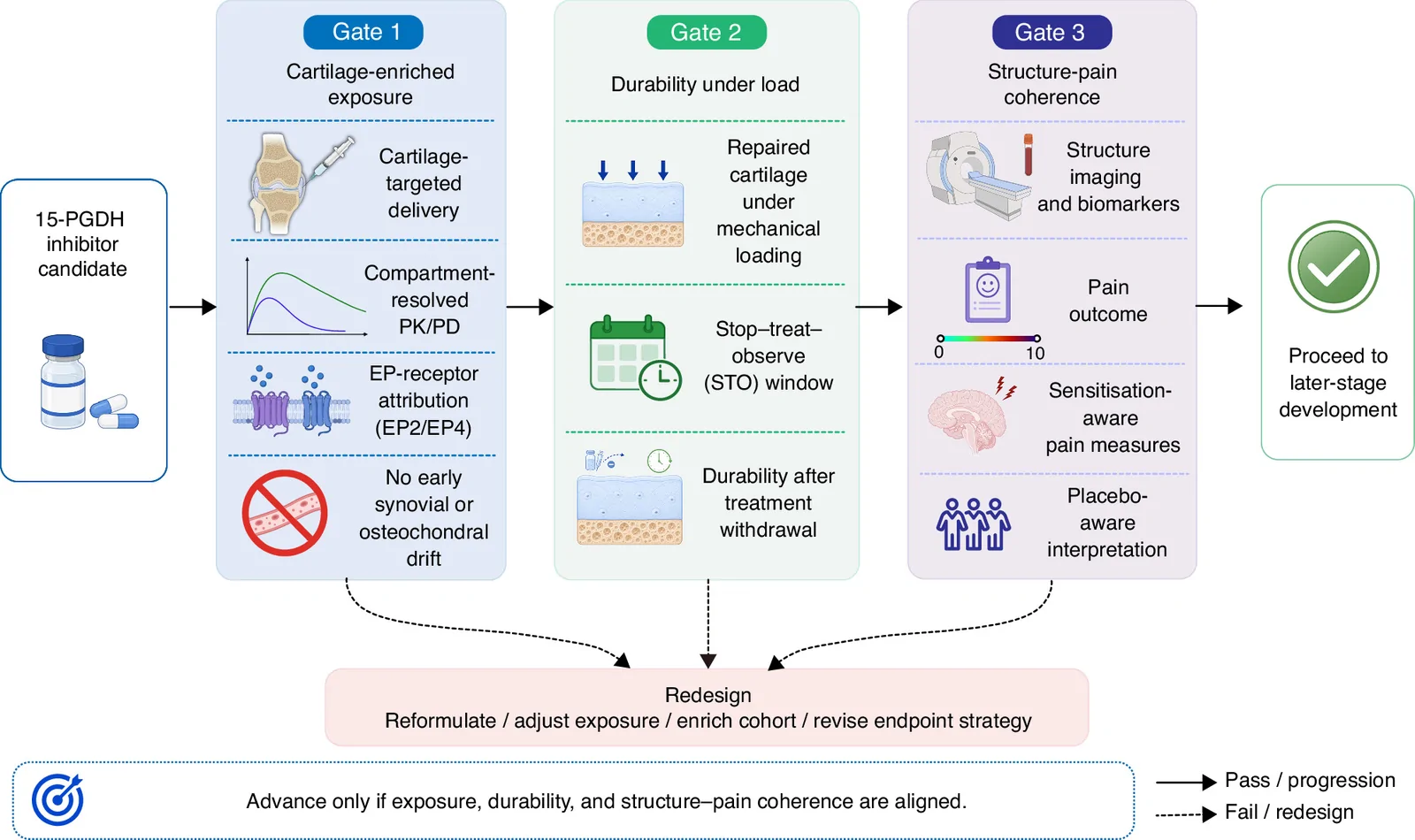
Translational checkpoints and decision gates for PGE_2_-stabilisation disease-modifying osteoarthritis strategies. A 15-PGDH inhibitor candidate should pass three linked gates before progression to later-stage development. Gate 1 tests cartilage-enriched exposure, including cartilage-targeted delivery, compartment-resolved PK/PD, EP-receptor attribution and absence of early synovial or osteochondral drift. Gate 2 tests durability under load, including repaired cartilage under mechanical loading, a stop–treat–observe durability window and persistence after treatment withdrawal. Gate 3 tests structure–pain coherence using structural imaging and biomarkers, pain outcomes, sensitisation-aware pain measures and placebo-aware interpretation. Failure at any gate should trigger redesign, including reformulation, exposure adjustment, cohort enrichment or endpoint revision, rather than simple dose escalation. Solid arrows indicate pass/progression; dashed arrows indicate fail/redesign

Progression to later-stage development should therefore require three evidence categories: cartilage-enriched engagement with receptor-proximal attribution and no early synovial or osteochondral drift; durability under mechanical loading, including after withdrawal; and pre-specified structure–pain coherence robust to placebo and sensitisation confounds. Only after these conditions are met can 15-PGDH inhibition be judged as a credible candidate for progression from murine regeneration to a human DMOAD strategy. An overview of these checkpoints and associated decision criteria is provided in Fig. 4.

## Development implications and trial strategy

OA heterogeneity makes mechanism-matched stratification central to interpreting both efficacy and liability. Clinical trajectories reflect the combined influence of synovitis, osteochondral remodelling, systemic milieu and pain-system plasticity.^1,27^ For PGE_2_ stabilisation, this heterogeneity becomes a pharmacological problem: because the intervention prolongs an endogenous and pleiotropic mediator, the benefit–risk profile will reflect each patient’s dominant joint compartment and pain phenotype, and is unlikely to be captured by cohort-level averages in heterogeneous trial populations.^4,5^

An endotype–exposure–endpoint alignment strategy translates this principle into trial design. Such a strategy would enrol patients in whom cartilage repair biology is plausible, bias exposure towards cartilage rather than liability compartments, and use endpoints that detect both benefit and drift. Prospectively specified axes could include structural stage, effusion–synovitis burden, subchondral activity (bone marrow lesions and neurovascular features) and pain phenotype (nociceptive-dominant versus sensitisation-enriched).^5,11^ Emerging stratification frameworks and large-scale genomics support feasibility by mapping OA to pathway-informative signals and clinically tractable subgroups.^10^

Selective receptor-bias mitigation should be treated as a contingency option rather than a default adjunct. If biomarker and imaging data indicate liability-dominant EP4 signalling in synovium, subchondral bone or nociceptive pathways, then locally restricted or time-limited EP4 modulation could be explored. Even then, current evidence cautions against treating EP4 as uniformly harmful: veterinary OA experience with grapiprant supports analgesic pathway selectivity,^42^ whereas both EP4 antagonism and EP4 agonism have shown chondroprotective effects in different experimental contexts.^41,43^ Any EP4-modulation strategy should therefore proceed only with receptor-resolved evidence that cartilage-reparative signalling is preserved.

### Early clinical strategy: demonstrating compartment-managed target engagement

In humans, the initial priority is to determine whether cartilage-relevant PGE_2_ signalling can be stabilised without synovial or osteochondral drift, a test that should precede any long-term structural claim.^13^ Early phase 1b/2a studies should therefore pair compartment-resolved pharmacokinetics/pharmacodynamics (PK/PD) with liability-proximal monitoring under matched dosing and timing.

Synovial fluid is an informative sampling compartment because it integrates signals from cartilage, synovium, fat pad and subchondral bone.^5^ Human atlases provide mechanistic context: if synovium and IPFP behave as an immunostromal unit with disease-linked programmes, synovial readouts may preferentially report the compartment that responds to stabilised PGE_2_ even when therapeutic intent is cartilage.^31^

Osteochondral surveillance should function as a go/no-go readout, not an exploratory add-on. The osteochondral junction is increasingly recognised as a pain-relevant interface where neurovascular remodelling sustains symptoms and shapes progression.^24^ Single-cell work suggests that BMLs are inflammatory microenvironments with senescence-associated signatures and plausible communication routes to cartilage damage.^53^ Early warning signals may therefore include BML dynamics, subchondral neurovascular markers and related osteochondral readouts. A pragmatic early-phase panel could include magnetic resonance imaging-quantified effusion–synovitis and BML trajectories, cartilage compositional measures where feasible, and pain outcomes that capture sensitisation rather than relying solely on a single scalar score.^4^

Dose selection should prioritise the spatiotemporal window of activity rather than exposure escalation alone. If the mechanism is chondrocyte state biasing rather than cell replacement,^13^ durability should be tested early: STO designs and biomarker trajectories provide a direct test of whether a transient signalling perturbation converts into stable tissue-level change. Post-traumatic OA prevention frameworks are informative because they use longitudinal joint-biology tracking and clarify the distinction between episodic symptom modulation and disease modification.^94^

### Positioning in the DMOAD landscape

Trial experience has shown that endpoint design is critical. Pain may improve through systemic risk-factor modification rather than joint repair; the STEP 9 semaglutide trial in obesity-associated knee OA illustrates symptomatic and functional improvement via sustained weight loss without implying cartilage regeneration.^70^

Intra-articular programmes can also yield signals that depend on baseline severity and endpoint choice; post-hoc analyses of lorecivivint datasets underscore conditional outcomes driven by enrolment and readout selection.^71^ For PGE_2_ stabilisation, these realities are compounded by two persistent challenges: large placebo/expectancy effects^7^ and frequent structure–symptom discordance shaped by central processing.^6^

Development should therefore begin with a clear, testable positioning strategy. Progression should be gated by evidence of: (i) compartment-managed target engagement consistent with cartilage-relevant biology; (ii) no early synovial or osteochondral drift that predicts liability; and (iii) a pain signal directionally coherent with joint biology rather than dominated by sensitisation or contextual variance. This evidentiary threshold is higher than demonstrating movement in pain or structure alone, but it is the most defensible route to a DMOAD claim in a field repeatedly derailed by discordance.^1,27^

**Fig. 5:**
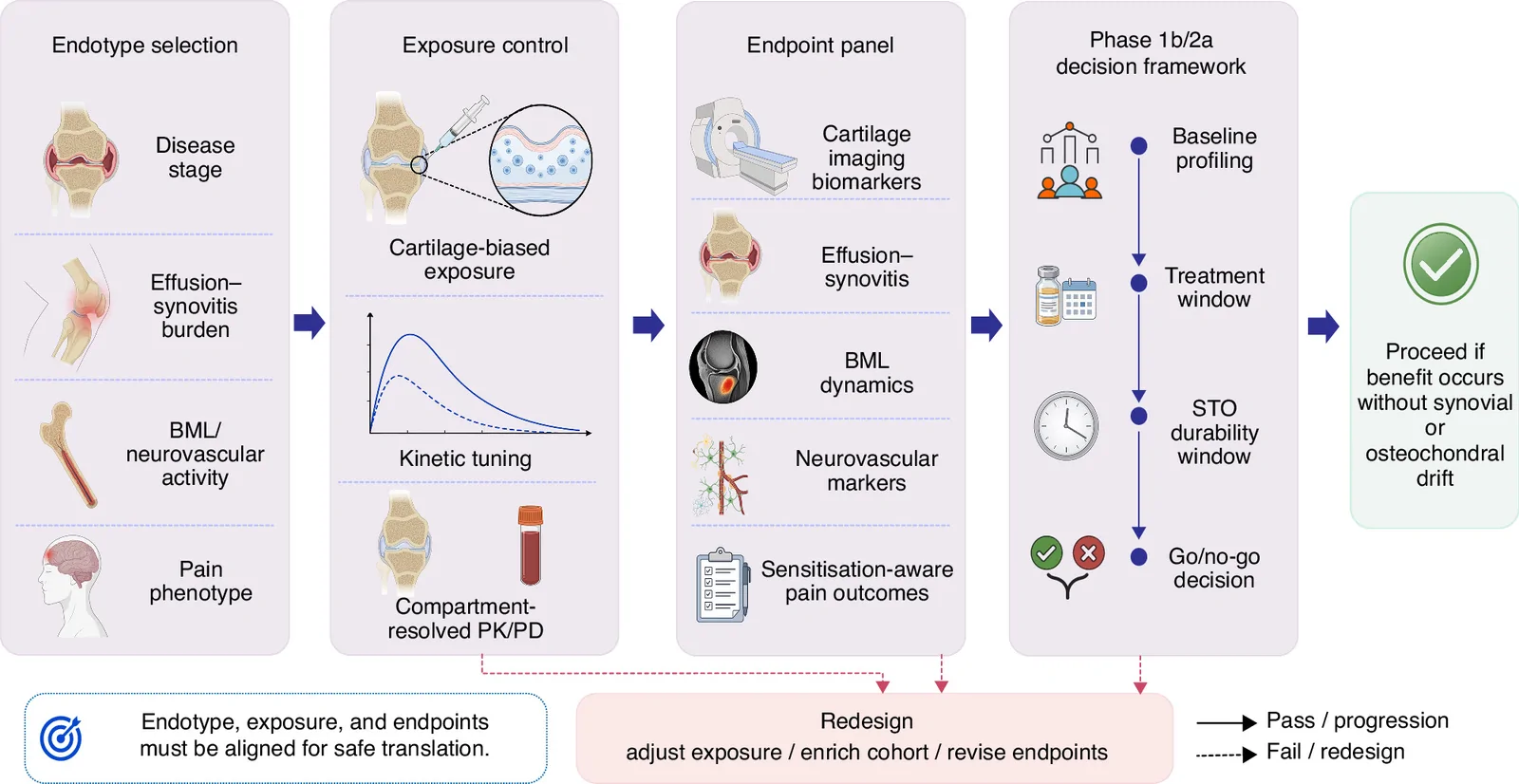
Endotype–exposure–endpoint alignment for early clinical development of 15-PGDH inhibitors. This early-phase framework aligns patient selection, exposure control, endpoint architecture and go/no-go decisions. Endotype selection considers disease stage, effusion–synovitis burden, bone marrow lesion or neurovascular activity and pain phenotype. Exposure control aims for cartilage-biased exposure through kinetic tuning and compartment-resolved PK/PD. The endpoint panel combines cartilage imaging biomarkers, effusion–synovitis assessment, bone marrow lesion dynamics, neurovascular markers and sensitisation-aware pain outcomes. The phase 1b/2a decision framework integrates baseline profiling, a treatment window, a stop–treat–observe durability window and pre-specified go/no-go decisions. Progression is supported only when benefit occurs without synovial or osteochondral drift; failure should trigger exposure adjustment, cohort enrichment or endpoint revision. Solid arrows indicate pass/progression; dashed arrows indicate fail/redesign

If these conditions are met, 15-PGDH inhibition could provide a stringent test of whether exposure control, liability surveillance and endpoint design can convert pleiotropic prostaglandin biology into a credible human disease-modifying strategy. Endotype–exposure–endpoint alignment and an early-phase progression framework are summarised in Fig. 5.

## Conclusion and outlook

This Review argues that 15-PGDH inhibition in OA should be understood as modulation of PGE_2_ skeletal interoception, not simply as a cartilage-intrinsic method for increasing PGE_2_. By slowing PGE_2_ catabolism, 15-PGDH inhibition can extend a signal that is read locally by chondrocytes and through EP4-positive sensory afferents, central circuits and sympathetic outflow. This provides a plausible explanation for why cartilage repair and reduced pain behaviours may occur together in mice, while making compartment control essential.

The translational issue is therefore where the extended PGE_2_ pulse occurs, how long it persists and which receptor programmes interpret it. A cartilage-biased or osteochondral-interface-biased signal may support matrix restoration and restrain excessive sympathetic norepinephrine output. A prolonged signal in synovium or subchondral bone may instead amplify inflammation, osteoclast-vascular-nerve coupling and pain-system sensitisation. Clinical development will therefore require compartment-resolved pharmacokinetics/pharmacodynamics, receptor-proximal attribution and early surveillance for synovial, osteochondral, interoceptive and pain-related liabilities.

Taken together, 15-PGDH inhibition should be regarded as a mechanistically plausible but still early DMOAD strategy. The revised interoception-centred framework clarifies both the rationale and the boundary conditions: the aim is to preserve reparative PGE_2_ skeletal interoception while preventing persistent liability-dominant PGE_2_ signalling outside cartilage. Progress on cartilage-enriched exposure, mechanical durability and structure–pain coherence will determine whether this approach can move from a preclinical observation to a credible translational programme in OA.

## Data Availability

No new data were generated or analysed in this review. All the information was derived from publicly available sources cited in the manuscript.

## References

[1] Collins, K. H., Haugen, I. K., Neogi, T. & Guilak, F. Osteoarthritis as a systemic disease. *Nat. Rev. Rheumatol.* **22**, 105–117 (2026). 10.1038/s41584-025-01332-8 PMC13556116 41339496

[2] Kloppenburg, M., Namane, M. & Cicuttini, F. Osteoarthritis. *Lancet* **405**, 71–85 (2025). 10.1016/S0140-6736(24)02322-5 39755397

[3] Muthu, S. et al. Failure of cartilage regeneration: emerging hypotheses and related therapeutic strategies. *Nat. Rev. Rheumatol.* **19**, 403–416 (2023). 10.1038/s41584-023-00979-5 37296196

[4] Batchelor, V., Perry, T. A., Cader, M. Z. & Vincent, T. L. Peripheral neuronal sensitization and neurovascular remodelling in osteoarthritis pain. *Nat. Rev. Rheumatol.* **21**, 526–545 (2025). 10.1038/s41584-025-01280-3 40796685

[5] Peters, H., Rockel, J. S., Little, C. B. & Kapoor, M. Synovial fluid as a complex molecular pool contributing to knee osteoarthritis. *Nat. Rev. Rheumatol.* **21**, 447–464 (2025). 10.1038/s41584-025-01271-4 40624394

[6] Barroso, J., Branco, P. & Apkarian, A. V. The causal role of brain circuits in osteoarthritis pain. *Nat. Rev. Rheumatol.* **21**, 261–274 (2025). 10.1038/s41584-025-01234-9 40164779

[7] Neogi, T. & Colloca, L. Placebo effects in osteoarthritis: implications for treatment and drug development. *Nat. Rev. Rheumatol.* **19**, 613–626 (2023). 10.1038/s41584-023-01021-4 PMC10615856 37697077

[8] GBD 2021 Osteoarthritis Collaborators Global, regional, and national burden of osteoarthritis, 1990–2020 and projections to 2050: a systematic analysis for the Global Burden of Disease Study 2021. *Lancet Rheumatol.* **5**, e508–e522 (2023). 10.1016/S2665-9913(23)00163-7 PMC10477960 37675071

[9] Zhang, B., Chen, Y., Chen, Q. & Zhang, H. Exosomal miRNAs in muscle-bone crosstalk: mechanistic links, exercise modulation and implications for sarcopenia, osteoporosis and osteosarcopenia. *Metabolism* **170**, 156333 (2025). 10.1016/j.metabol.2025.156333 40550325

[10] Hatzikotoulas, K. et al. Translational genomics of osteoarthritis in 1 962 069 individuals. *Nature* **641**, 1217–1224 (2025). 10.1038/s41586-025-08771-z PMC12119359 40205036

[11] Fuggle, N. R. et al. Novel approaches to the stratified management of knee osteoarthritis. *Nat. Rev. Rheumatol.* **21**, 684–695 (2025). 10.1038/s41584-025-01305-x 41044417

[12] Yao, Q. et al. Osteoarthritis: pathogenic signaling pathways and therapeutic targets. *Signal Transduct. Target. Ther.* **8**, 56 (2023). 10.1038/s41392-023-01330-w PMC9898571 36737426

[13] Singla, M. et al. Inhibition of 15-hydroxy prostaglandin dehydrogenase promotes cartilage regeneration. *Science* **391**, 1053–1062 (2026). 10.1126/science.adx6649 PMC13127300 41308124

[14] Pandey, A. & Bhutani, N. Profiling joint tissues at single-cell resolution: advances and insights. *Nat. Rev. Rheumatol.* **20**, 7–20 (2024). 10.1038/s41584-023-01052-x PMC11674069 38057475

[15] Lv, X., Gao, F. & Cao, X. Skeletal interoception in bone homeostasis and pain. *Cell Metab.* **34**, 1914–1931 (2022). 10.1016/j.cmet.2022.09.025 PMC9742337 36257317

[16] Wang, Z. et al. Hypothalamus regulates anabolic metabolism of articular cartilage superficial chondrocytes through PGE2 skeletal interoception. *Adv. Sci. (Weinh.)* **12**, e2501039 (2025). 10.1002/advs.202501039 PMC12097074 40138204

[17] Gao, F. et al. Brain regulates weight-bearing bone through PGE2 skeletal interoception: implication of ankle osteoarthritis and pain. *Bone Res.* **12**, 16 (2024). 10.1038/s41413-024-00316-w PMC10914853 38443372

[18] Chen, H. et al. Prostaglandin E2 mediates sensory nerve regulation of bone homeostasis. *Nat. Commun.* **10**, 181 (2019). 10.1038/s41467-018-08097-7 PMC6331599 30643142

[19] Yang, D., Xu, J., Xu, K. & Xu, P. Skeletal interoception in osteoarthritis. *Bone Res.* **12**, 22 (2024). 10.1038/s41413-024-00328-6 PMC10985098 38561376

[20] Guan, Z. et al. Sympathetic innervation induces exosomal miR-125 transfer from osteoarthritic chondrocytes, disrupting subchondral bone homeostasis and aggravating cartilage damage in aging mice. *J. Adv. Res.* **69**, 245–260 (2025). 10.1016/j.jare.2024.03.022 PMC11954818 38554999

[21] Huang, W. et al. Small molecule inhibitors of 15-PGDH exploit a physiologic induced-fit closing system. *Nat. Commun.* **14**, 784 (2023). 10.1038/s41467-023-36463-7 PMC9922282 36774348

[22] Müller, E., Kruse, B. & Böttcher, J. P. Prostaglandins in cancer revisited: principles of production, mechanisms of immune regulation, and therapeutic perspectives. *Blood* **147**, 713–724 (2026). 10.1182/blood.2025029806 41288533

[23] Jiang, W. et al. PGE2 activates EP4 in subchondral bone osteoclasts to regulate osteoarthritis. *Bone Res.* **10**, 27 (2022). 10.1038/s41413-022-00201-4 PMC8904489 35260562

[24] Malfait, A. M. & Obeidat, A. M. Targeting osteoarthritis where it hurts: the osteochondral junction. *Nat. Rev. Rheumatol.* **21**, 319–320 (2025). 10.1038/s41584-025-01257-2 PMC12355372 40263460

[25] Rubino, M. et al. Inhibition of eicosanoid degradation mitigates fibrosis of the heart. *Circ. Res.* **132**, 10–29 (2023). 10.1161/CIRCRESAHA.122.321475 36475698

[26] Xia, Z. et al. Structure and transport mechanism of the human prostaglandin transporter SLCO2A1. *Nat. Commun.* **16**, 8124 (2025). 10.1038/s41467-025-63615-8 PMC12398505 40885756

[27] Tang, S. et al. Osteoarthritis. *Nat. Rev. Dis. Prim.* **11**, 10 (2025). 10.1038/s41572-025-00594-6 39948092

[28] Hansen, C. E. et al. Spatial mapping of the AA–PGE2–EP axis in multiple sclerosis lesions. *Acta Neuropathol.* **149**, 39 (2025). 10.1007/s00401-025-02878-3 PMC12041062 40299057

[29] Villa, M. et al. Prostaglandin E2 controls the metabolic adaptation of T cells to the intestinal microenvironment. *Nat. Commun.* **15**, 451 (2024). 10.1038/s41467-024-44689-2 PMC10781727 38200005

[30] Fan, X. et al. Spatial analysis of the osteoarthritis microenvironment: techniques, insights, and applications. *Bone Res.* **12**, 7 (2024). 10.1038/s41413-023-00304-6 PMC10838951 38311627

[31] Tang, S. et al. Single-cell atlas of human infrapatellar fat pad and synovium implicates APOE signaling in osteoarthritis pathology. *Sci. Transl. Med.* **16**, eadf4590 (2024). 10.1126/scitranslmed.adf4590 38266107

[32] Raut, R. D. et al. A multi-tissue human knee single-cell atlas identifies that osteoarthritis reduces regenerative tissue stem cells while increasing inflammatory pain macrophages. *Commun. Biol.* **8**, 1146 (2025). 10.1038/s42003-025-08586-8 PMC12317986 40753276

[33] Gahbauer, S. et al. Docking for EP4R antagonists active against inflammatory pain. *Nat. Commun.* **14**, 8067 (2023). 10.1038/s41467-023-43506-6 PMC10700596 38057319

[34] Zhang, J. et al. Osteocyte connexin hemichannels and prostaglandin E2 release dictate bone marrow mesenchymal stromal cell commitment. *Proc. Natl. Acad. Sci. USA* **122**, e2412144122 (2025). 10.1073/pnas.2412144122 PMC11848350 39937859

[35] Yang, D., Xu, K., Xu, X. & Xu, P. Revisiting prostaglandin E2: a promising therapeutic target for osteoarthritis. *Clin. Immunol.* **260**, 109904 (2024). 10.1016/j.clim.2024.109904 38262526

[36] Neogi, T., Dell’Isola, A., Englund, M. & Turkiewicz, A. Frequent use of prescription NSAIDs among people with knee or hip osteoarthritis despite contraindications to or precautions with NSAIDs. *Osteoarthr. Cartil.* **32**, 1628–1635 (2024). 10.1016/j.joca.2024.07.010 PMC11563888 39068982

[37] Hasvik, E., Gran, J. M., Haugen, A. J. & Grøvle, L. Strategies to manage auxiliary pain medications in chronic pain trials: a topical review. *Eur. J. Clin. Pharmacol.* **78**, 1377–1384 (2022). 10.1007/s00228-022-03355-6 PMC9365733 35842457

[38] Borst, J. M. et al. Placebo effect sizes in clinical trials of knee osteoarthritis using intra-articular injections of biologic agents. *Arthritis Care Res. (Hoboken)* **77**, 998–1006 (2025). 10.1002/acr.25526 PMC12307098 40105673

[39] Edwards, R. R. et al. Optimizing and accelerating the development of precision pain treatments for chronic pain: IMMPACT review and recommendations. *J. Pain.* **24**, 204–225 (2023). 10.1016/j.jpain.2022.08.010 PMC10868532 36198371

[40] Robertson-Plouch, C. et al. A randomized clinical efficacy study targeting mPGES1 or EP4 in dogs with spontaneous osteoarthritis. *Sci. Transl. Med.* **11**, eaaw9993 (2019). 10.1126/scitranslmed.aaw9993 31666405

[41] Jin, Y. et al. A novel prostaglandin E receptor 4 (EP4) small molecule antagonist induces articular cartilage regeneration. *Cell Discov.* **8**, 24 (2022). 10.1038/s41421-022-00382-6 PMC8901748 35256606

[42] Rausch-Derra, L., Huebner, M., Wofford, J. & Rhodes, L. A prospective, randomized, masked, placebo-controlled multisite clinical study of grapiprant, an EP4 prostaglandin receptor antagonist (PRA), in dogs with osteoarthritis. *J. Vet. Intern. Med.* **30**, 756–763 (2016). 10.1111/jvim.13948 PMC4913586 27075237

[43] Murahashi, Y. et al. Oral administration of EP4-selective agonist KAG-308 suppresses mouse knee osteoarthritis development through reduction of chondrocyte hypertrophy and TNF secretion. *Sci. Rep.* **9**, 20329 (2019). 10.1038/s41598-019-56861-6 PMC6937271 31889132

[44] Lacher, S. B. et al. PGE2 limits effector expansion of tumour-infiltrating stem-like CD8+ T cells. *Nature* **629**, 417–425 (2024). 10.1038/s41586-024-07254-x PMC11078747 38658748

[45] Wu, Y. et al. Structural insights into selective and dual antagonism of EP2 and EP4 prostaglandin receptors. *EMBO J.* **44**, 7242–7262 (2025). 10.1038/s44318-025-00611-0 PMC12669672 41162752

[46] Baas, J. D., Varga, J., Feghali-Bostwick, C., Peters-Golden, M. & Fortier, S. M. Distinct cAMP regulation in scleroderma lung and skin myofibroblasts governs their dedifferentiation via p38α inhibition. *FASEB J.* **39**, e70762 (2025). 10.1096/fj.202500694RR PMC12186599 40552889

[47] Wang, N. et al. Mechanotransduction pathways in articular chondrocytes and the emerging role of estrogen receptor-α. *Bone Res.* **11**, 13 (2023). 10.1038/s41413-023-00248-x PMC9984452 36869045

[48] Sato, T. et al. Prostaglandin EP2 receptor signalling inhibits the expression of matrix metalloproteinase 13 in human osteoarthritic chondrocytes. *Ann. Rheum. Dis.* **70**, 221–226 (2011). 10.1136/ard.2009.118620 20870807

[49] Schmidt, S., Klampfleuthner, F. A. M., Renkawitz, T. & Diederichs, S. Cause and chondroprotective effects of prostaglandin E2 secretion during mesenchymal stromal cell chondrogenesis. *Eur. J. Cell Biol.* **103**, 151412 (2024). 10.1016/j.ejcb.2024.151412 38608422

[50] Lohse, M. J., Bock, A. & Zaccolo, M. G protein-coupled receptor signaling: new insights define cellular nanodomains. *Annu. Rev. Pharmacol. Toxicol.* **64**, 387–415 (2024). 10.1146/annurev-pharmtox-040623-115054 37683278

[51] Bai, Z. et al. Synovial fibroblast gene expression is associated with sensory nerve growth and pain in rheumatoid arthritis. *Sci. Transl. Med.* **16**, eadk3506 (2024). 10.1126/scitranslmed.adk3506 PMC11931728 38598614

[52] Sokhi, U. K. et al. Opposing regulation of TNF responses by IFN-γ and a PGE2–cAMP axis that is apparent in rheumatoid and immune checkpoint inhibitor-induced arthritis human IL-1β^+^ macrophages. *eLife* **14**, RP104367 (2025). 10.7554/eLife.104367 PMC12263154 40662950

[53] Lou, P. et al. Single-cell sequencing reveals a senescent immune landscape in bone marrow lesions inducing articular cartilage damage in osteoarthritis. *Bone Res.* **13**, 94 (2025). 10.1038/s41413-025-00467-4 PMC12639131 41272390

[54] Boyer, T. L. et al. Cartilage targeting cationic peptide carriers display deep cartilage penetration and retention in a rabbit model of post-traumatic osteoarthritis. *Osteoarthr. Cartil.* **33**, 721–734 (2025). 10.1016/j.joca.2025.04.001 PMC12242911 40222626

[55] Deng, R. et al. Chondrocyte membrane-coated nanoparticles promote drug retention and halt cartilage damage in rat and canine osteoarthritis. *Sci. Transl. Med.* **16**, eadh9751 (2024). 10.1126/scitranslmed.adh9751 38381849

[56] Bracho-Sanchez, E. et al. Suppression of local inflammation via galectin-anchored indoleamine 2,3-dioxygenase. *Nat. Biomed. Eng.* **7**, 1156–1169 (2023). 10.1038/s41551-023-01025-1 PMC10504068 37127708

[57] Fan, Y. et al. Unveiling inflammatory and prehypertrophic cell populations as key contributors to knee cartilage degeneration in osteoarthritis using multi-omics data integration. *Ann. Rheum. Dis.* **83**, 926–944 (2024). 10.1136/ard-2023-224420 PMC11187367 38325908

[58] Matta, C. et al. Insights into chondrocyte populations in cartilaginous tissues at the single-cell level. *Nat. Rev. Rheumatol.* **21**, 465–477 (2025). 10.1038/s41584-025-01275-0 40640375

[59] Li, J. et al. Synovium and infrapatellar fat pad share common mesenchymal progenitors and undergo coordinated changes in osteoarthritis. *J. Bone Miner. Res.* **39**, 161–176 (2024). 10.1093/jbmr/zjad009 PMC11323896 38477740

[60] Scerif, F. & Eldridge, S. E. Osteoarthritis year in review 2025: biology. *Osteoarthr. Cartil.* **34**, 213–220 (2026). 10.1016/j.joca.2025.12.013 41418993

[61] Bergman, R. F. et al. Sexual dimorphism of the synovial transcriptome underpins greater PTOA disease severity in male mice following joint injury. *Osteoarthr. Cartil.* **32**, 1060–1073 (2024). 10.1016/j.joca.2023.07.012 37716404

[62] Geraghty, T. et al. Age-associated changes in knee osteoarthritis, pain-related behaviors, and dorsal root ganglia immunophenotyping of male and female mice. *Arthritis Rheumatol.* **75**, 1770–1780 (2023). 10.1002/art.42530 PMC10543384 37096632

[63] Bolander, J. et al. The synovial environment steers cartilage deterioration and regeneration. *Sci. Adv.* **9**, eade4645 (2023). 10.1126/sciadv.ade4645 PMC10121162 37083524

[64] Palma, C. et al. A compartmentalized joint-on-chip (JoC) model to unravel the contribution of cartilage and synovium to osteoarthritis pathogenesis. *Adv. Sci. (Weinh.)* **12**, e00374 (2025). 10.1002/advs.202500374 PMC12622499 40936111

[65] Dvir-Ginzberg, M., Maatuf, Y. H. & Mobasheri, A. Do we understand sex-related differences governing dimorphic disease mechanisms in preclinical animal models of osteoarthritis? *Osteoarthr*. *Cartil* **32**, 1054–1057 (2024). 10.1016/j.joca.2024.03.113 38531464

[66] Fu, W. et al. NaV1.7 as a chondrocyte regulator and therapeutic target for osteoarthritis. *Nature* **625**, 557–565 (2024). 10.1038/s41586-023-06888-7 PMC10794151 38172636

[67] Karsdal, M. A. et al. Reflections from the OARSI 2022 clinical trials symposium: the pain of OA—deconstruction of pain and patient-reported outcome measures for the benefit of patients and clinical trial design. *Osteoarthr. Cartil.* **31**, 1293–1302 (2023). 10.1016/j.joca.2023.06.006 PMC11184959 37380011

[68] Reihs, E. et al. Beyond symptomatic alignment: evaluating the integration of causal mechanisms in matching animal models with human pathotypes in osteoarthritis research. *Arthritis Res. Ther.* **27**, 109 (2025). 10.1186/s13075-025-03561-4 PMC12084918 40382623

[69] Zhao, L., Lai, Y., Jiao, H. & Huang, J. Nerve growth factor receptor limits inflammation to promote remodeling and repair of osteoarthritic joints. *Nat. Commun.* **15**, 3225 (2024). 10.1038/s41467-024-47633-6 PMC11018862 38622181

[70] Bliddal, H. et al. Once-weekly semaglutide in persons with obesity and knee osteoarthritis. *N. Engl. J. Med.* **391**, 1573–1583 (2024). 10.1056/NEJMoa2403664 39476339

[71] Tambiah, J., Kennedy, S., Swearingen, C., McAlindon, T. & Yazici, Y. Impact of structural severity on outcomes in knee osteoarthritis: an analysis of data from phase 2 and phase 3 lorecivivint clinical trials. *Rheumatol. (Oxf.)* **64**, 2583–2590 (2025). 10.1093/rheumatology/keae610 39495154

[72] Yazici, Y. et al. A phase 3, 28-week, multicentre, randomised, double-blind, placebo-controlled trial (OA-10) to evaluate the efficacy and safety of a single injection of lorecivivint in the target knee joint of moderately to severely symptomatic osteoarthritis patients. *Clin. Exp. Rheumatol.* **43**, 847–853 (2025). 10.55563/clinexprheumatol/gskbin 39808288

[73] Previtali, D., Boffa, A., Di Laura Frattura, G., Merli, G. & Filardo, G. Placebo response to intra-articular injections in knee osteoarthritis: magnitude, evolution over time, and influencing factors. A systematic review and meta-analysis with meta-regression. *EFORT Open Rev.* **10**, 782–795 (2025). 10.1530/EOR-2025-0022 PMC12495556 41031623

[74] Zhou, K. et al. A “best-in-class” systemic biomarker predictor of clinically relevant knee osteoarthritis structural and pain progression. *Sci. Adv.* **9**, eabq5095 (2023). 10.1126/sciadv.abq5095 PMC9876540 36696492

[75] Hansen, R. T., Chenu, C., Sofat, N. & Pitsillides, A. A. Bone marrow lesions: plugging the holes in our knowledge using animal models. *Nat. Rev. Rheumatol.* **19**, 429–445 (2023). 10.1038/s41584-023-00971-z 37225964

[76] Vincent, T. L. & Miller, R. E. Molecular pathogenesis of OA pain: past, present, and future. *Osteoarthr. Cartil.* **32**, 398–405 (2024). 10.1016/j.joca.2024.01.005 PMC10984780 38244717

[77] Nordberg, R. C. et al. Recent advancements in cartilage tissue engineering innovation and translation. *Nat. Rev. Rheumatol.* **20**, 323–346 (2024). 10.1038/s41584-024-01118-4 PMC11524031 38740860

[78] Zhang, C., Vedadghavami, A., He, T., Charles, J. F. & Bajpayee, A. G. Cationic carrier mediated delivery of anionic contrast agents in low doses enable enhanced computed tomography imaging of cartilage for early osteoarthritis diagnosis. *ACS Nano* **17**, 6649–6663 (2023). 10.1021/acsnano.2c12376 PMC10629240 36989423

[79] Kim, J. S. et al. FDA/Arthritis Foundation osteoarthritis drug development workshop recap: assessment of long-term benefit. *Semin. Arthritis Rheum.* **56**, 152070 (2022). 10.1016/j.semarthrit.2022.152070 PMC9452453 35870222

[80] Bakooshli, M. A. et al. Regeneration of neuromuscular synapses after acute and chronic denervation by inhibiting the gerozyme 15-prostaglandin dehydrogenase. *Sci. Transl. Med.* **15**, eadg1485 (2023). 10.1126/scitranslmed.adg1485 PMC10763629 37820010

[81] Koh, Y. et al. Inhibiting 15-PGDH blocks blood-brain barrier deterioration and protects mice from Alzheimer’s disease and traumatic brain injury. *Proc. Natl. Acad. Sci. USA* **122**, e2417224122 (2025). 10.1073/pnas.2417224122 PMC12130856 40397680

[82] Wang, Y. et al. Methotrexate to treat hand osteoarthritis with synovitis (METHODS): an Australian, multisite, parallel-group, double-blind, randomised, placebo-controlled trial. *Lancet* **402**, 1764–1772 (2023). 10.1016/S0140-6736(23)01572-6 37839420

[83] Jiang, T. et al. Bidirectional association identified between synovitis and knee and hand osteoarthritis: a general population-based study. *Lancet Reg. Health West. Pac.* **50**, 101169 (2024). 10.1016/j.lanwpc.2024.101169 PMC11372600 39234534

[84] Zhang, S. et al. Remodeling articular immune homeostasis with an efferocytosis-informed nanoimitator mitigates rheumatoid arthritis in mice. *Nat. Commun.* **14**, 817 (2023). 10.1038/s41467-023-36468-2 PMC9925448 36781864

[85] Hasegawa, T. et al. Macrophages and nociceptor neurons form a sentinel unit around fenestrated capillaries to defend the synovium from circulating immune challenge. *Nat. Immunol.* **25**, 2270–2283 (2024). 10.1038/s41590-024-02011-8 PMC11588661 39587345

[86] Nassini, R. et al. Targeting prostaglandin E2 receptor 2 in Schwann cells inhibits inflammatory pain but not inflammation. *Nat. Commun.* **16**, 8262 (2025). 10.1038/s41467-025-63782-8 PMC12462433 40998803

[87] Schulz, J. M. et al. Changes and associations between synovial fluid and magnetic resonance imaging markers of osteoarthritis after high tibial osteotomy. *Arthritis Res. Ther.* **26**, 176 (2024). 10.1186/s13075-024-03409-3 PMC11465750 39390512

[88] Sun, Q. et al. Peripheral nerves in the tibial subchondral bone: the role of pain and homeostasis in osteoarthritis. *Bone Jt. Res.* **11**, 439–452 (2022). 10.1302/2046-3758.117.BJR-2021-0355.R1 PMC9350689 35775136

[89] Tu, M. et al. Inhibition of cyclooxygenase-2 activity in subchondral bone modifies a subtype of osteoarthritis. *Bone Res.* **7**, 29 (2019). 10.1038/s41413-019-0071-x PMC6804921 31666999

[90] Kantarci, H. et al. Schwann cell-secreted PGE2 promotes sensory neuron excitability during development. *Cell* **187**, 4690–4712.e30 (2024). 10.1016/j.cell.2024.07.033 PMC11967275 39142281

[91] Joshi, N. et al. A mechanically resilient soft hydrogel improves drug delivery for treating post-traumatic osteoarthritis in physically active joints. *Proc. Natl. Acad. Sci. USA* **122**, e2409729122 (2025). 10.1073/pnas.2409729122 PMC12002200 40163719

[92] Qin, W. et al. Neurovascularization inhibiting dual responsive hydrogel for alleviating the progression of osteoarthritis. *Nat. Commun.* **16**, 1390 (2025). 10.1038/s41467-025-56727-8 PMC11799281 39910066

[93] Diamond, L. E., Grant, T. & Uhlrich, S. D. Osteoarthritis year in review 2023: biomechanics. *Osteoarthr. Cartil.* **32**, 138–147 (2024). 10.1016/j.joca.2023.11.015 38043858

[94] Kraus, V. B. & Hsueh, M. F. Molecular biomarker approaches to prevention of post-traumatic osteoarthritis. *Nat. Rev. Rheumatol.* **20**, 272–289 (2024). 10.1038/s41584-024-01102-y 38605249

